# Direct interpretation of the X-ray and neutron three-dimensional difference pair distribution functions (3D-ΔPDFs) of yttria-stabilized zirconia

**DOI:** 10.1107/S205252062300121X

**Published:** 2023-02-24

**Authors:** Ella Mara Schmidt, Reinhard B. Neder, James D. Martin, Arianna Minelli, Marie-Hélène Lemée, Andrew L. Goodwin

**Affiliations:** a Faculty of Geosciences, MARUM and MAPEX, University of Bremen, Bremen, Germany; b Inorganic Chemistry Laboratory, University of Oxford, Oxford, United Kingdom; c Institute of Condensed Matter Physics, Friedrich-Alexander University, Erlangen, Germany; d Department of Chemistry, North Carolina State University, Rayleigh, USA; e Institut Laue-Langevin, Grenoble, France; University of Geneva, Switzerland

**Keywords:** diffuse scattering, cubic zirconia, three-dimensional difference pair distribution function (3D-ΔPDF)

## Abstract

Three-dimensional difference pair distribution functions (3D-ΔPDFs) from X-ray and neutron diffraction experiments are reported for yttria-stabilized zirconia (Zr_0.82_Y_0.18_O_1.91_). A quantitative analysis of the signatures in the three-dimensional difference pair distribution functions is used to establish that oxygen ions neighbouring a vacancy shift by 0.525 (5) Å along 〈1, 0, 0〉 towards the vacancy while metal ions neighbouring a vacancy shift by 0.465 (2) Å along 〈1, 1, 1〉 away from the vacancy. The neutron 3D-ΔPDF shows a tendency for vacancies to cluster along 〈½, ½, ½〉, which results in sixfold coordinated metal ions.

## Abstract 2

Three-dimensional difference pair distribution functions (3D-ΔPDFs) from X-ray and neutron diffraction experiments are used to identify local stabilization mechanisms in yttria-stabilized cubic zirconia.

## 1. Introduction

The technological importance of zirconia (ZrO_2_) is undoubted and this is underlined by its numerous applications in the ceramic industry. At ambient pressure and temperature ZrO_2_ is monoclinic (*P*2_1_/*c*) but upon increasing temperature it transforms to a tetragonal phase (*P*4_2_/*nmc*, at approximately 1440 K) and a cubic phase ( 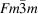 , at approximately 2640 K) (Boysen *et al.*, 1991 ▸; Bondars *et al.*, 1995 ▸). The addition of aliovalent oxides (CaO, MgO or Y_2_O_3_) allows the cubic phase to be stabilized at ambient conditions and results in a material that is widely applied because of its strength, high refractive index and thermal-shock resistance (Stapper *et al.*, 1999 ▸). To maintain the overall charge balance in cubic stabilized zirconia (CSZ), oxygen vacancies are introduced into the structure which makes CSZ an ion conductor used in solid oxygen fuel cells (Tsampas *et al.*, 2015 ▸), oxygen sensors (Schindler *et al.*, 1989 ▸) and oxygen pumps (Pham & Glass, 1998 ▸).

**Figure 1:**
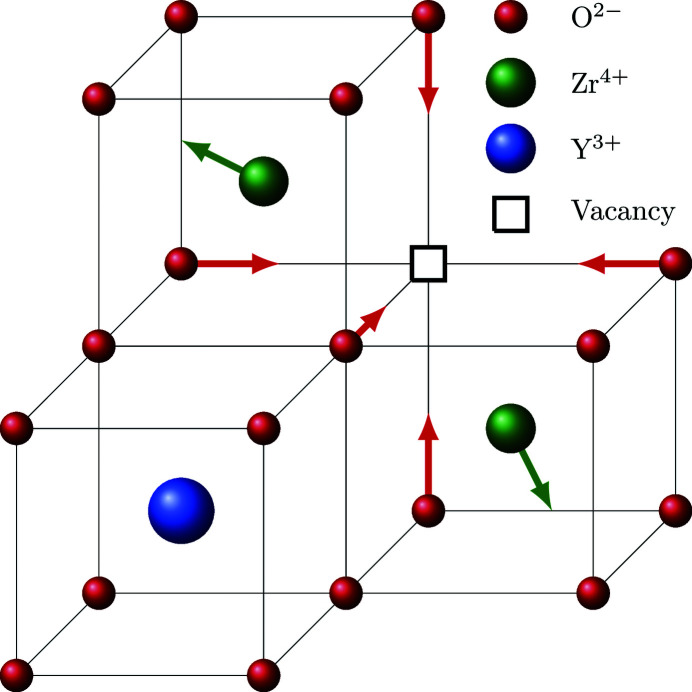
Schematics of local relaxations in YSZ as reported in the literature (Frey *et al.*, 2005 ▸; Khan *et al.*, 1998 ▸; Fèvre *et al.*, 2005 ▸). Oxygen ions (red) relax along 〈1, 0, 0〉 towards a neighbouring vacancy, next neighbour (NN) Zr ions relax along 〈1, 1, 1〉 away from the vacancy, while next nearest neighbour (NNN) dopant ions do not show significant relaxations.

The high-temperature cubic polymorph of pure ZrO_2_ adopts the fluorite structure, where each Zr^4+^ is in regular eightfold coordination. Upon cooling, the local coordination environment in pure ZrO_2_ is distorted and Zr^4+^ is sevenfold coordinated. On average, CSZ still adapts the fluorite structure but the introduction of oxygen vacancies reduces the average coordination number to 8 − *x*, where *x* depends on the dopant ion charge and concentration. The resulting complex defect structure of CSZ is a long-standing problem that has been addressed by numerous computational and experimental means. The nature of the most localized interactions is widely agreed upon in the literature and reproduced in Fig. 1 ▸ (Frey *et al.*, 2005 ▸; Khan *et al.*, 1998 ▸; Fèvre *et al.*, 2005 ▸): oxygen ions neighbouring a vacancy relax along 〈1, 0, 0〉 towards this vacancy. Electrostatic effects and atomic sizes need to be taken into account when evaluating which type of metal ions neighbours a vacancy. Whether the oxygen vacancies are located preferably as a nearest neighbour (NN, *i.e.* in the coordination of the dopant metal ion) or a next nearest neighbour (NNN) depends on the concentration, size and charge of the dopant ion (Khan *et al.*, 1998 ▸). For yttria-stabilized zirconia (YSZ), simulations show that NNN vacancies are preferred (Bogicevic & Wolverton, 2003 ▸; Khan *et al.*, 1998 ▸). The Zr^4+^ ions next to the vacancy relax along 〈1, 1, 1〉 away from the vacancy (Frey *et al.*, 2005 ▸; Khan *et al.*, 1998 ▸; Fèvre *et al.*, 2005 ▸). The exact magnitude of the relaxations and correlated further neighbour displacements depend on type and concentration of the dopant ion and only a limited agreement is reported in literature [see Frey *et al.* (2005 ▸) for a review].

In our contribution we focus on the defect structure in YSZ because of its technological importance as an oxygen ion conductor. We use three-dimensional difference pair distribution functions (3D-ΔPDFs) from neutron and X-ray diffraction experiments to quantify local-order principles. The material under investigation is by no means a newly studied material because YSZ was first described in 1951 by Hund (1951 ▸). Previous theoretical and experimental investigations include first-principle calculations (Stapper *et al.*, 1999 ▸), molecular dynamics simulations (Bogicevic & Wolverton, 2003 ▸; Fabris *et al.*, 2002 ▸), Bragg data analysis (Kaiser-Bischoff *et al.*, 2005 ▸; Ishizawa *et al.*, 1999 ▸; Morinaga *et al.*, 1979 ▸), extended X-ray absorption fine structure (EXAFS) (Ishizawa *et al.*, 1999 ▸; Catlow *et al.*, 1986 ▸; Veal *et al.*, 1988 ▸), nuclear magnetic resonance (NMR) measurements (Viefhaus & Müller, 2006 ▸; Kim *et al.*, 2007 ▸) and single-crystal diffuse scattering analysis (Andersen *et al.*, 1986 ▸; Welberry *et al.*, 1992 ▸, 1995 ▸; Goff *et al.*, 1999 ▸). The latter has so far been restricted to selected layers in reciprocal space. More recent computational and technological advances allow the collection and interpretation of full three-dimensional data (Welberry *et al.*, 2003 ▸), opening up the possibility to re-visit controversially discussed defect clusters by utilizing the highly powerful 3D-ΔPDF technique (Weber & Simonov, 2012 ▸; Roth & Iversen, 2019 ▸; Simonov *et al.*, 2014 ▸), which represents differences in interatomic vector probabilities with respect to the average structure.

This work is organized as follows. We begin by presenting our experimentally obtained diffuse scattering data and quantitatively analyse the 3D-ΔPDFs for the dominant local interactions. We compare these results to literature findings and discuss the novel insights obtained by the 3D-ΔPDF analysis. Using our 3D-ΔPDF analysis we perform Monte Carlo simulations to show how the quantitative insight into local-order models can be realized in a model crystal. We conclude by discussing the significance of our quantitative 3D-ΔPDF analysis and outline the strengths of combining X-ray and neutron single-crystal diffuse measurements to solve complex local-order problems as encountered in YSZ.

## 2. Results and discussion

### 2.1. Sample material

The zirconia samples have a composition of Zr_0.82_Y_0.18_O_1.91_, grown by the skull melting method, delivered by Djevahirdjan S. A., Monthey, Switzerland. The composition was confirmed by EDX measurements (see supporting information). For neutron measurements, the large clear single crystals were cut with a diamond saw into cubes with an edge length of approximately 5 mm. For X-ray diffraction measurements, the larger crystals were mechanically ground to a diameter of about 150 µm and polished. All measurements were carried out under ambient conditions.

### 2.2. X-ray and neutron diffraction experiments

X-ray diffraction experiments were performed on a Rigaku Synergy S diffractometer equipped with an Eiger 1M detector using Mo radiation. To avoid possible fluorescence a threshold of 17.4 keV was set for the detector. Simple φ scans with 0.5° step width and 120 s exposure time were taken. 3D diffuse scattering data was reconstructed using the orientation matrix provided by *CrysAlis PRO* (Agilent, 2014 ▸) and custom Python scripts using *Meerkat* (Simonov, 2020 ▸). The experimentally obtained unit-cell parameter was *a* = 5.1505 (5) Å.

Neutron diffraction experiments were carried out at the D19 instrument (λ = 0.95 Å, 0.1° steps, 80 s exposure per frame) (ILL, Grenoble, France) utilizing a 180° φ scan. ILL are data available at https://dx.doi.org/10.5291/ILL-DATA.5-13-277. 3D diffuse scattering data reconstruction utilized the orientation matrix as provided by *Int3d* (Katcho *et al.*, 2021 ▸) and a custom Python script.

**Figure 2:**
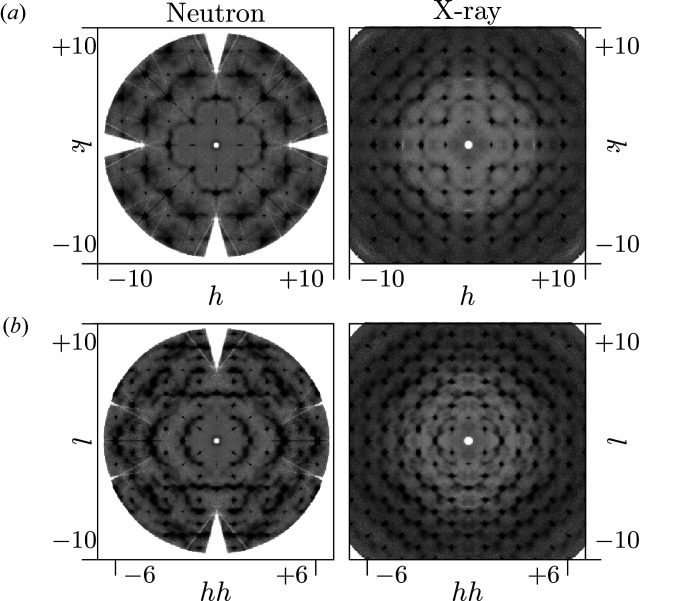
Reconstructed and symmetry-averaged diffuse scattering of YSZ obtained from neutron (left) and X-ray (right) diffraction experiments: (*a*) the *hk*0 layer and (*b*) the *hhl* layer.

The reflection conditions for space group 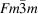 were fulfilled in all cases and after careful inspection the data were symmetry averaged for 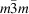 Laue symmetry. Symmetry-averaged reciprocal space maps of selected layers are presented in Fig. 2 ▸.

### 2.3. 3D-ΔPDF maps

The general data processing procedure to obtain 3D-ΔPDF experiments is described by Koch *et al.* (2021 ▸). The experimentally obtained data were treated with the KAREN outlier rejection algorithm (Weng *et al.*, 2020 ▸). Additionally a custom punch-and-fill approach that interpolates the intensity in punched voxels was used to eliminate residual Bragg intensities. To avoid Fourier ripples the data were multiplied with a Gaussian falloff that smooths the edges of the measured reciprocal space section [see Weng *et al.* (2020) ▸]. The FFT algorithm as implemented in *Meerkat* (Simonov, 2020 ▸) was used to obtain 3D-ΔPDF maps.

**Figure 3:**
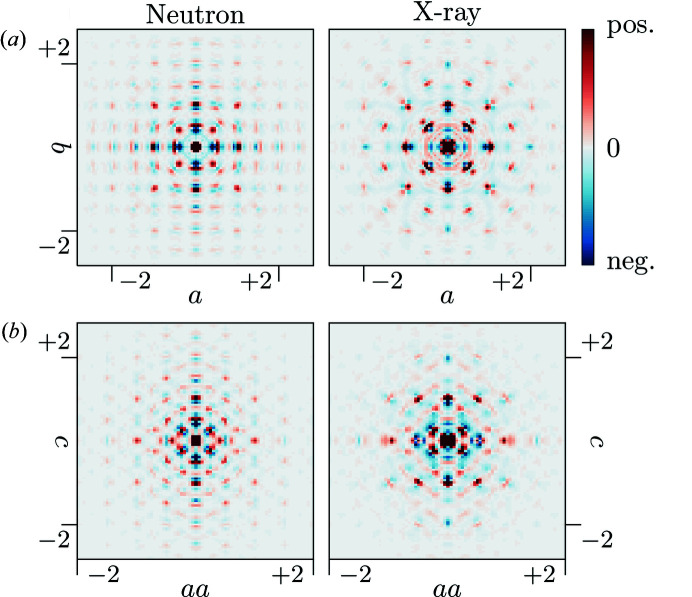
3D-ΔPDF maps of YSZ obtained from neutron (left) and X-ray (right) diffraction experiments: (*a*) the *ab*0 layer and (*b*) the *aac* layer. Positive intensities in red, negative intensities in blue.

The 3D-ΔPDF maps in the *ab*0 layer and the *aac* layer are shown in Fig. 3 ▸. All 3D-ΔPDFs show a variety of different signatures. The most pronounced features are observed at the shortest interatomic vectors indicating strong local-order principles, which we will analyse in the following sections.

### 2.4. Oxygen nearest neighbour interactions

**Figure 4:**
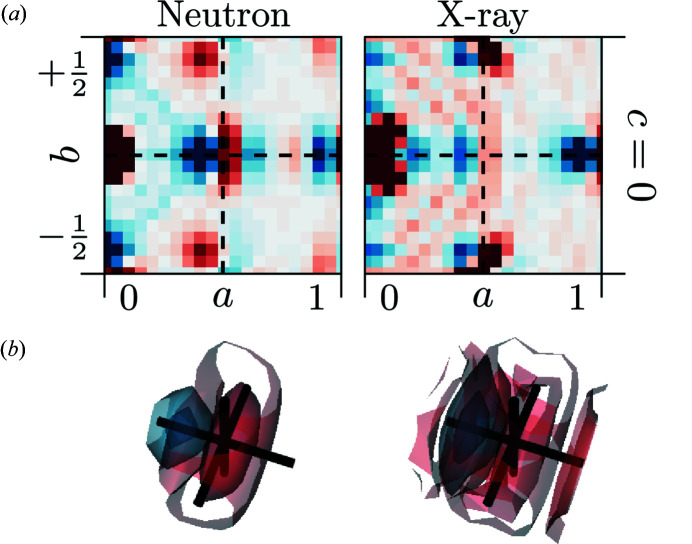
3D-ΔPDFs of YSZ obtained from neutron (left) and X-ray (right) diffraction experiments around the 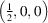 interatomic vector. (*a*) Two-dimensional section in the *ab*0 layer. (*b*) Three-dimensional rendering of the intensity distribution. Volume shown in the region 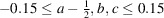 . Black lines indicate the average structure interatomic vector at 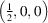 . 3D renderings are on the same relative intensity scale with respect to the minimum intensity in the rendering section. Positive intensities in red, negative intensities in blue.

The 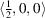 interatomic vectors are the shortest interatomic vectors in the idealized zirconia structure that only occur in the oxygen substructure. Detailed two-dimensional sections and three-dimensional renderings of the 3D-ΔPDFs around the (½ ,0, 0) interatomic vector are shown in Fig. 4 ▸.

For the neutron diffraction experiments, a clear minimum shifted to shorter interatomic vectors by 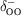 at 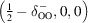 and a clear maximum shifted to longer interatomic vectors by 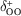 at 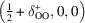 can be observed. In the X-ray diffraction experiment the observed trend is the same but much weaker due to the much lower scattering power of oxygen as compared to the heavier metal ions. This is the typical signature for a size-effect like relaxation (Weber & Simonov, 2012 ▸). A minimum in the 3D-ΔPDF indicates less scattering density than suggested by the average structure model, a maximum indicates more scattering density. The 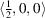 interatomic vectors discussed here only occur in the oxygen substructure, hence only correlations that include oxygen ions and vacancies need to be considered. Any real structure configuration that includes a vacancy has zero scattering density, while any real structure configuration that involves two oxygen ions has more scattering density than suggested by the average structure (*b*_O_ × *b*_O_ > 0.955*b*_O_ × 0.955*b*_O_). Hence we can attribute the minimum observed here to real structure configurations that involve a vacancy and occur with a higher probability at shorter interatomic vectors, while configurations that involve two oxygen ions can be attributed to the maximum and occur with a higher probability at longer interatomic vectors. This is a clear indication that locally oxygen ions relax towards neighbouring vacancies along the 〈1, 0, 0〉 directions.

This finding is consistent with computational and experimental reports from the literature, and qualitatively displayed in Fig. 1  ▸(Frey *et al.*, 2005 ▸; Khan *et al.*, 1998 ▸; Fèvre *et al.*, 2005 ▸). The MD simulations (on Zr_0.9375_Y_0.0675_O_1.96875_) by Fabris *et al.* (2002 ▸) report a relaxation of oxygen ions neighbouring a vacancy of 0.27 Å, the MD simulations (on Zr_0.865_Y_0.135_O_1.9325_) of Fèvre *et al.* (2005 ▸) report a shift of 0.40 Å and the first-principle calculations (on Zr_0.9375_Y_0.0675_O_1.96875_) of Stapper *et al.* (1999 ▸) report a shift of 0.24 Å. Experimental reports using Bragg data refinements from Goff *et al.* (1999 ▸) (on Zr_0.8_Y_0.2_O_1.9_, shift 0.04 r.l.u. ≈ 0.20 Å), Kaiser-Bischoff *et al.* (2005 ▸) (on Zr_0.74_Y_0.26_O_1.87_, shift 0.24 Å) and Ishizawa *et al.* (1999 ▸) (on Zr_0.758_Y_0.242_O_1.879_, shift 0.31 Å) report similar results. These results show no clear indication of a correlation of the shift magnitude and the dopant concentration of the sample. Our results also support a clear shift of the oxygen ions along 〈1, 0, 0〉, and show no indication for previously suggested shifts along 〈1, 1, 1〉 directions (Ishizawa *et al.*, 1999 ▸; Argyriou *et al.*, 1996 ▸) as such a shift would distort the homogeneous maximum at 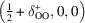 . However, an additional, more isotropic off-axis relaxation is possible, as the observed maximum in the 3D-ΔPDF shows a disc-like feature. This indicates that for two oxygen ions that are separated by 
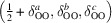
 , 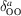 shows a much more narrow distribution than 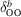 and 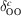 .

The 3D-ΔPDF analysis also allows a quantitative estimation of the shift magnitudes. For this purpose we fit the position of the minimum centred at 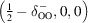 with a three-dimensional Gaussian distribution. The resulting parameter for 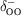 is 1.02 (1) × 10^−1^ r.l.u. for the fit to the neutron data and 0.91 (1) × 10^−1^ r.l.u. for the fit to the X-ray data. From electrostatic considerations we assume that neighbouring vacancies are highly unlikely. Therefore we interpret the minimum as generated by real structure oxygen–vacancy correlations. With the experimentally obtained unit-cell parameter *a* = 5.1505 (5) Å and our refined shift magnitude we estimate that the oxygen ions neighbouring a vacancy relax 0.525 (5) Å along 〈1, 0, 0〉 towards the vacancy. The shift amplitude is larger than previous experimental reports that utilized Bragg data analysis suggested and more similar to the MD simulations of Fèvre *et al.* (2005 ▸). Due to the much higher relative sensitivity of the neutron 3D-ΔPDF we used the parameter derived from the neutron data in the shift estimation. Nevertheless, the X-ray 3D-ΔPDF shows a clear signature at 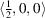 and the quantitative analysis suggests that oxygen displacements can be directly observed in X-ray diffraction experiments, with a reasonable quantitative agreement to the neutron data.

### 2.5. Oxygen metal interactions

**Figure 5:**
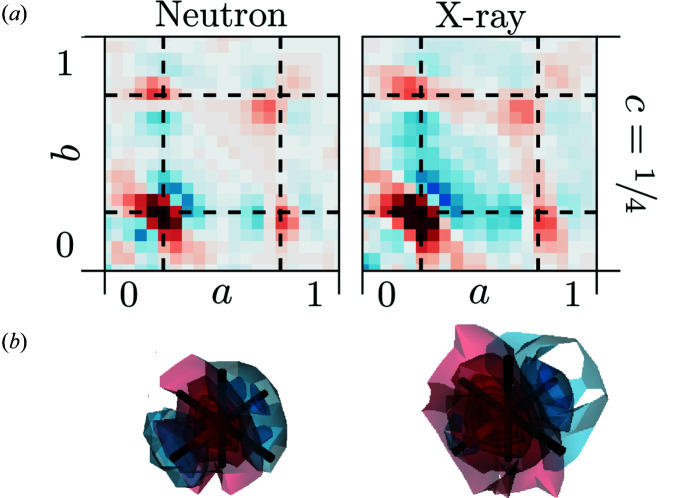
3D-ΔPDFs obtained from neutron (left) and X-ray (right) diffraction experiments. (*a*) Two-dimensional section in the *ab*0.25 layer. (*b*) Three-dimensional rendering of the intensity distribution. Volume shown in the region 0.1 ≤ *a*, *b*, *c* ≤ 0.40. Black lines indicate the average interatomic vector at 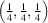 . Positive intensities in red, negative intensities in blue.

The 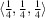 interatomic vectors only occur between the metal and the oxygen substructures. Detailed two- and three-dimensional 3D-ΔPDFs of the 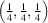 interatomic vector are shown in Fig. 5 ▸ and resemble the typical signature for a positive atomic displacement parameter (ADP) correlation where the local bond distance variation is smaller than suggested by the ADPs of the average structure. The simple analysis and interpretation of interatomic vector as for the 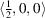 vector is not possible here, as there are four distinct pair correlations that can be observed at the 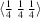 inter­atomic vectors: possible pairs are Zr–O, Zr–vacancy, Y–O and Y–vacancy.

For X-ray diffraction, there is negligible contrast between Zr^4+^ and Y^3+^. For neutron diffraction the contrast between Zr and Y is not large either [*b*(Zr) = 7.16 versus *b*(Y) = 7.75], which does not allow for a direct disentanglement of signatures associated with Y–O and Zr–O distances. However, similar to the 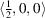 interatomic vectors, any real space configuration that involves a vacancy has zero scattering density. Therefore, positive correlations indicate interatomic vectors with a higher probability for locally observed metal–oxygen vectors. In a similar fashion to the 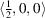 interatomic vectors we fit the position of the maximum with a three-dimensional Gaussian distribution centred at 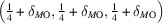 . The resulting parameters for δ_*M*O_ are −1.58 (10) × 10^−2^ r.l.u. for X-ray and −0.95 (8) × 10^−2^ r.l.u. for neutron diffraction. The observed maximum in the 3D-ΔPDF is shifted towards the centre of the 3D-ΔPDF space, contracted in the direction of the shift and elongated perpendicular of to the shift. This indicates a local contraction of the oxygen–metal bond length, which can be achieved if metal ions relax away from neighbouring vacancies along the 〈1, 1, 1〉 directions. The resulting local configurations show a variety of oxygen–metal bond distances, which accounts for the elongation of the maximum perpendicular to the shift direction. With the experimentally obtained unit-cell parameter *a* = 5.1505 (5) Å, we estimate the average metal–O^2−^ nearest neighbour distance at 2.09 (1) Å for X-ray and 2.15 (1) Å for neutron diffraction experiments. The differences in the calculated distances can be attributed to the differences in scattering contrasts. The fact that Y has the larger neutron scattering length and the estimated average metal–oxygen bond length is longer for the neutron refinement than for the X-ray refinement indicate that locally Y—O bond lengths are larger than Zr—O bond length, which is consistent with ionic radii (Shannon, 1976 ▸; Prince, 2004 ▸).

The arrangement of the oxygen ions and the vacancies in the oxygen substructure determines the coordination numbers of the metals. In the average structure of cubic ZrO_2_, each Zr^4+^ ion is in regular cubic coordination, while monoclinic ZrO_2_ only shows sevenfold coordination (Frey *et al.*, 2005 ▸). Y_2_O_3_ shows sixfold coordinated metal ions at ambient conditions (Antic *et al.*, 1993 ▸). Hence, it seems that both the Zr and the Y ions compete for lower coordination numbers; electrostatic effects and atomic sizes need to be taken into account when evaluating which type of metal ions neighbours a vacancy in the real structure. The simulations of Bogicevic & Wolverton (2003 ▸) and Khan *et al.* (1998 ▸) suggest that for YSZ NNN vacancies are preferred. Experimentally this is confirmed by the ^89^Y solid-state MAS-NMR studies of Viefhaus & Müller (2006 ▸), which only show a significant onset of the peaks for six- and sevenfold coordinated Y ions above 10 mol% Y_2_O_3_, which is above the 9 mol% Y_2_O_3_ in our specimen.

Different metal coordination and different ionic radii lead to different oxygen–metal bond lengths. In Table 1 ▸ we summarize simulated bond distances given by Khan *et al.* (1998 ▸), experimentally measured bond distances from EXAFS studies in YSZ by Ishizawa *et al.* (1999 ▸) and Catlow *et al.* (1986 ▸) and the sum of effective ionic radii based on Shannon (1976 ▸). However, the presence of the oxygen vacancies and the associated relaxations are likely to yield not perfect coordination polyhedra but rather distorted bonding environments (see Fig. 1 ▸). Such distorted bond environments are also supported by density functional theory calculations on superstructures of Zr_1−*x*_Y_*x*_O_2−*x*/2_ (Pruneda & Artacho, 2005 ▸), X-ray absorption studies (Li *et al.*, 1993 ▸) and the fact that sevenfold coordination in pure ZrO_2_ at room temperature shows a considerable variance in Zr^4+^—O^2−^ bond distances (between 2.05 Å and 2.26 Å) (Yashima *et al.*, 1995 ▸).

The estimated average metal–oxygen nearest neighbour distances from our 3D-ΔPDF analysis is, therefore, likely also an average obtained from several distorted bond environments. Comparing the deduced average distance with the sum of the atomic radii (Shannon, 1976 ▸) in Table 1 ▸ a sevenfold Zr coordination is supported. This is consistent with the EXAFS studies by Ishizawa *et al.* (1999 ▸) and Catlow *et al.* (1986 ▸), the ^89^Y solid-state MAS-NMR studies of Viefhaus & Müller (2006 ▸), as well as the computational work of Bogicevic & Wolverton (2003 ▸) and Khan *et al.* (1998 ▸) that favour dopant ions as NNN to the vacancies, which results in a preference for Zr in sevenfold coordination.

### 2.6. Metal–metal interactions

**Figure 6:**
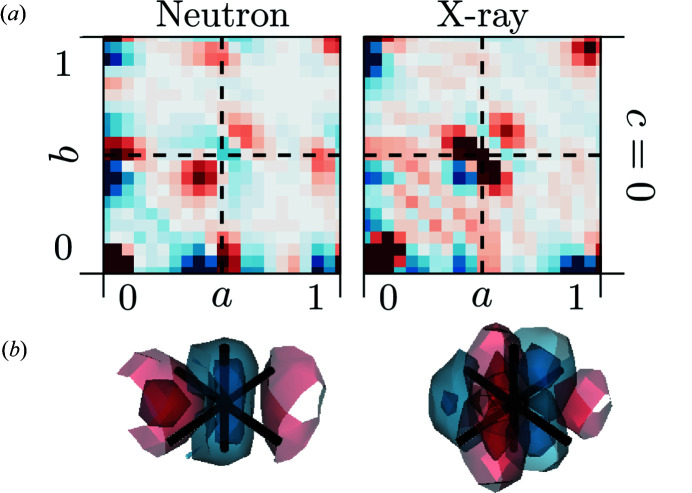
3D-ΔPDFs obtained from neutron (left) and X-ray (right) diffraction experiments. (*a*) Two-dimensional plot in the *ab*0 layer. (*b*) Three-dimensional rendering of the intensity distribution. Volume shown in the region 0.25 ≤ *a*, *b*, *c* + 0.5 ≤ 0.75. Black lines indicate the average interatomic vector at 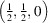 . Positive intensities in red, negative intensities in blue.

The 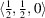 are the most complex of the nearest neighbour interatomic vectors as they occur in both structures. The maxima associated with O–O correlations are overlaid by metal–metal interactions. 3D-ΔPDFs of the 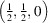 inter­atomic vector are shown in Fig. 6 ▸.

For both our diffraction experiments, the observed signatures are complex and the differences highlight the contrast between X-ray and neutron diffraction experiments. However, both signatures show two distinct locations of maxima, which can be interpreted as a result of different local configurations: configurations where both or one of the bridging oxygen ions are missing in between the metals will result in a different metal–metal interatomic vector than those of configurations where both bridging oxygen ions are present. Furthermore the exact intensity and distance distribution will depend on the coordination numbers and types of metals involved. The entanglement of the interatomic distances involved in the signature at the 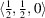 vectors is therefore effectively a multi body correlation and can only be accessed indirectly with scattering experiments where only pair correlations can be probed directly (Welberry, 2004 ▸; Baake & Grimm, 2009 ▸). This limitation is also present in 3D-ΔPDF and a direct interpretation of the signature at 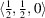 relies on information deduced from shorter interatomic vectors.

However, assuming a shift of the metal ions neighbouring a vacancy away from the vacancy, as deduced from our data in the previous section, we can assign the maximum observed at 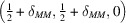 : for simplicity we consider a single pair of metal ions *M*1 at (0, 0, 0) and *M*2 at 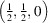 with the bridging vacancy located at 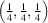 . The metal ions will relax away from the vacancies in the 〈1, 1, 1〉 directions. *M*1 will be shifted to (−Δ_*M*O_, − Δ_*M*O_, − Δ_*M*O_) and *M*2 will be shifted to 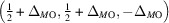 , yielding the new inter-atomic vector between *M*1 and *M*2 as 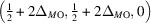 . While the shift δ_*M*O_ determined in the previous section yields an average metal–oxygen bond length, the shift Δ_*M*O_ we determine here is directly related to the shift of a metal atom neighbouring a vacancy. We quantify this shift by fitting the maximum at 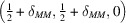 in the X-ray 3D-ΔPDF with a three-dimensional Gaussian distribution. We choose the X-ray data for this estimation as the scattering contributions here are dominated by the contributions of the metal ions. The fitted shift magnitude is δ_*MM*_ = 2Δ_*M*O_ = 1.043 (4) × 10^−1^ r.l.u. This corresponds to a metal shift along the 〈1, 1, 1〉 directions away from the vacancy of 0.465 (2) Å, which is slightly more pronounced than references in the literature. The MD simulations of Fabris *et al.* (2002 ▸) (Zr_0.9375_Y_0.0675_O_1.96875_) report a metal shift away from the neighbouring vacancy of 0.18 Å, the MD simulations of Fèvre *et al.* (2005 ▸) (Zr_0.865_Y_0.135_O_1.9325_) report a shift of 0.1 Å and the first-principle calculations of Stapper *et al.* (1999 ▸) (Zr_0.9375_Y_0.0675_O_1.96875_) report a shift of 0.18 Å. Experimental reports using Bragg data refinements from Goff *et al.* (1999 ▸) (Zr_0.8_Y_0.2_O_1.9_, shift of 0.028 r.l.u. ≈ 0.11 Å), Kaiser-Bischoff *et al.* (2005 ▸) (Zr_0.74_Y_0.26_O_1.87_, shift 0.22 Å) and Ishizawa *et al.* (1999 ▸) (Zr_0.758_Y_0.242_O_1.879_, 0.0219 r.l.u. shift 0.08 Å) vary largely in the reported shift magnitude and show no clear indication of a correlation of the shift magnitude and the dopant concentration of the sample.

### Possibility of vacancy clustering and interstitial metal atoms at 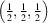


2.7.

**Figure 7:**
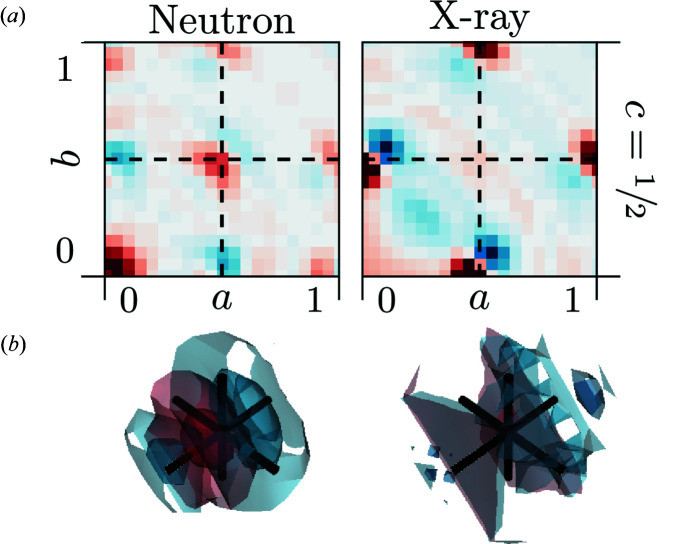
3D-ΔPDFs obtained from neutron (left) and X-ray (right) diffraction experiments. (*a*) Two-dimensional plot in the *ab*0.5 layer. (*b*) Three-dimensional rendering of the intensity distribution. Volume shown in the region 0.25 ≤ *a*, *b*, *c* ≤ 0.75. Black lines indicate the average interatomic vector at 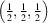 . Positive intensities in red, negative intensities in blue.

Questions that have been discussed controversially in the literature are the possibility of vacancy clusters along the 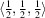 vectors – eventually forming chains of pyrochlore-like structural elements (Welberry *et al.*, 1993 ▸; Goff *et al.*, 1999 ▸) – and the possibility of interstitial metal ions at 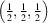 (Goff *et al.*, 1999 ▸). Both of these questions can be clarified by analysing the 3D-ΔPDFs in the vicinity of 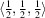 (see Fig. 7 ▸).

The possibility of metal ion interstitials at 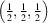 as considered by Goff *et al.* (1999 ▸) can also be ruled out here by the absence of relevant signatures in the X-ray 3D-ΔPDF in Fig. 7 ▸. Possible interstitial metal ions at 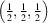 would lead to metal–metal interatomic vectors at 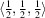 , which are not described by our average structural model and would hence result in a signature in the 3D-ΔPDF. The lower scattering power of oxygen compared to the metal ions – supported by the relative strength of the signatures in the X-ray 3D-ΔPDFs observed at 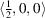 and 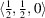 – suggests that the X-ray 3D-ΔPDF would be very sensitive to such a signature, while in the neutron case the signature present at 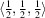 can be interpreted in terms of oxygen–oxygen correlations.

The shortest oxygen–oxygen interatomic vectors are 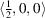 and 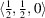 . For these vectors vacancy clustering becomes highly unlikely from an electrostatic viewpoint (Bogicevic & Wolverton, 2003 ▸). The shortest vectors where vacancy pairs are allowed from electrostatic considerations are the 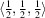 vectors. This yields the possibility to obtain six-coordinated metal ions in the structure. The doping level in the specimen at hand (Zr_0.82_Y_0.18_O_1.91_) is low enough to distribute the vacancies in the structure without needing to form sixfold coordinated ions, unlike for higher-doped variants such as described by Welberry *et al.* (1992 ▸). The in-depth simulation-based study of Bogicevic & Wolverton (2003 ▸), however, suggests that the formation of double vacancies along 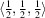 can nevertheless be favourable.

The signatures in Fig. 7 ▸ are very weak in the case of X-ray diffraction – basically indistinguishable from residual background noise. The situation is different for the neutron scattering case: here the scattering length of oxygen is comparable to that of the metals and the relative strength of signatures attributed ot oxygen–oxygen interactions is much greater than in the case of X-ray diffraction. Here, we observe a clear positive signature at 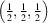 suggesting a positive correlation. If we consider pure substitutional disorder, positive correlations indicate a higher probability for likewise neighbours at this interatomic vector (Weber & Simonov, 2012 ▸). In our case this indicates a higher probability for oxygen–oxygen and vacancy–vacancy pairs along 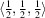 , *i.e.* a tendency to form double-vacancy pairs along this interatomic vector. Comparing the strength of the signature with the signature at 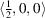 (note Figs. 4 ▸ and 7 ▸ are on the same relative scale), the signature of the double vacancies along 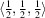 is much weaker. This suggests that there is no strict formation of double vacancies which would in turn lead to formation of zigzag chains of six-coordinated metal ions and local pyrochlore-like structural elements. This observation is in agreement with the findings of Welberry *et al.* (1995 ▸).

### 2.8. Longer-range interactions and extent of correlations

A direct interpretation of longer-range interactions in terms of underlying configurations is basically impossible due to the configurational complexity of possible arrangements of nearest neighbour, next nearest neighbour and next next nearest neighbour oxygen–vacancy pairs and metal–metal pairs. Nevertheless, the obtained 3D-ΔPDFs in Fig. 3 ▸ allow a direct judgement of the extent to which correlations due to the previously identified local correlations persist. For both the neutron and X-ray 3D-ΔPDF we do not observe correlations that are significantly longer than two unit cells, *i.e.* ≈ 10 Å, which is well below the estimated observable limit (see supporting information) and significantly shorter than previously reported in literature (Goff *et al.*, 1999 ▸; Frey *et al.*, 2005 ▸).

Possible differences in the neutron and X-ray 3D-ΔPDFs allow distinguishing whether the correlations are propagated in both substructures to the same extent: the limited sensitivity of X-ray 3D-ΔPDFs to oxygen–oxygen correlations allows the interpretation of the extent of correlations in terms of metal displacements only. In the X-ray 3D-ΔPDF, correlations along 〈1, 1, 0〉 persist longer than in the neutron case (see *e.g.* signature at 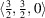 in Fig. 3 ▸), providing evidence that the metal–metal nearest neighbour distortion described in §2.6 is propagated to further neighbours in the same direction.

### 2.9. 3D-ΔPDF informed modelling

The detailed analysis of the experimentally obtained 3D-ΔPDFs we performed in the previous sections lays the groundwork for the simulation of a simplistic atomistic model that realizes the correlations we derived. For this purpose we use three successive Monte Carlo simulations, where the first two simulations establish the chemical ordering and the third simulation relaxes the atomistic positions according to our analysis. We simulate five model crystals of 10 × 10 × 10 unit cells. First the metal ions are distributed at random to match the average composition of 82% Zr and 18% Y ions. Charge balance is ensured by removing the suitable amount of oxygen ions.

The first Monte Carlo simulations induces chemical ordering of the metal ions. We assume for electrostatic reasons that Y ions tend to avoid being close to each other. The Monte Carlo simulations swap Zr and Y ions to avoid NN and NNN Y–Y pairs.

The second Monte Carlo simulation then swaps oxygen ions and vacancies in such a fashion that:

(i) Y-ions prefer eightfold coordination and avoid being next to vacancies,

(ii) Vacancy pairs separated by 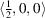 and 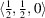 are penalized with a high energy,

(iii) A minimal energy gain is introduced for vacancies separated by 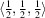 .

The resulting structures fulfil (i) and (ii) without violations. On average 3.06 (13)% of the metal ions are sixfold coordinated.

The third Monte Carlo simulation then introduces relaxations as deduced from the analysis of the 3D-ΔPDFs: assuming that every vacancy introduced in the structure causes its six neighbouring oxygen ions to relax towards the vacancy along the 〈1, 0, 0〉 directions and its four nearest neighbour metal ions to relax along 〈1, 1, 1〉 directions away from the vacancy, we introduced a static shift for the respective percentage of the ions and then used the Monte Carlo algorithm as implemented in the *DISCUS* program (Neder & Proffen, 2008 ▸) to switch the displacements. The energy targets were set as spring potentials with metal–oxygen target distances taken as the sum of the ionic radii (Shannon, 1976 ▸).

**Figure 8:**
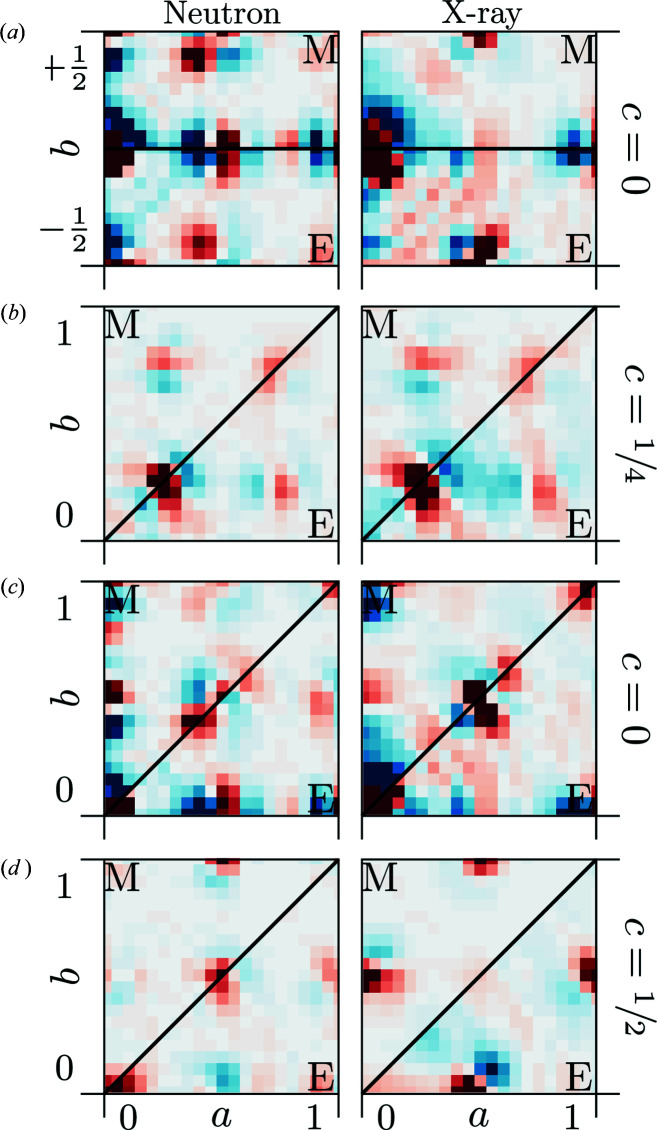
3D-ΔPDFs obtained from our simple atomistic model (M) from neutron (left) and X-ray (right) diffuse scattering calculations compared to the experimentally obtained 3D-ΔPDFs (E). (*a*) Two-dimensional section around 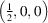 in the *ab*0 layer. Model top, experiment bottom. (*b*) Two-dimensional section around 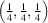 in the *ab*0.25 layer. Model top left, experiment bottom right. (*c*) Two-dimensional section around 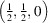 in the *ab*0 layer. Model top left, experiment bottom right. (*d*) Two-dimensional section around 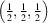 in the *ab*0.5 layer. Model top left, experiment bottom right.

The diffuse scattering (see supporting information) was calculated using the *DISCUS* program (Neder & Proffen, 2008 ▸) on a grid adapted to the size of the supercell. To obtain the model 3D-ΔPDFs we used a customized punch and fill algorithm (punch size was one voxel, as there is no experimental broadening of Bragg reflections in the calculated diffuse scattering). The same Gaussian falloff was multiplied to the simulated data as was to the experimental data and *Meerkat* (Simonov, 2020 ▸) was used to obtain the resulting simulated 3D-ΔPDFs in the regions of interest shown in Fig. 8 ▸ and in the supporting information.

A comparison of the 3D-ΔPDFs from our simple model and the respective sections in the experimentally obtained 3D-ΔPDFs in Fig. 8 ▸ shows that the nature of the major features that we quantified in our analysis are reproduced. The majority of the positions of maxima and minima are reproduced well while we observe more significant differences in the intensity distribution and the spatial extent of the features. These discrepancies we attribute to the simplicity of our model that only contains the quantities derived from a direct interpretation of the experiment. A refinement, *e.g.* using a reverse Monte Carlo simulation, would likely result in a much better agreement between data and model. However, our emphasis here is on the direct interpretation of the 3D-ΔPDF and our model confirms that the quantities we derive from this direct interpretation can be used to build a simple model that reproduces the nature of the local interactions and therefore confirms the results from our direct interpretation.

## 3. Concluding remarks

The defect structure of YSZ has been previously studied by several computational and experimental techniques (Frey *et al.*, 2005 ▸). Bragg data analysis, NMR and EXAFS studies provide only limited information about the local order in the system and a comprehensive model of the defect structure can only be obtained from single crystal diffuse scattering analysis. In the past, such analysis required computationally expensive modelling, and a direct interpretation of present distortions from the measured reciprocal space sections required very extensive expert knowledge or was simply impossible (Andersen *et al.*, 1986 ▸; Welberry *et al.*, 1993 ▸, 1995 ▸; Goff *et al.*, 1999 ▸). With the advances in experimental techniques it is now possible to obtain full three-dimensional reciprocal space coverage and the application of the 3D-ΔPDF allows direct interpretation of the data in terms of defect models (Weber & Simonov, 2012 ▸; Roth & Iversen, 2019 ▸; Simonov *et al.*, 2014 ▸).

In our contribution, we demonstrated how to combine the information from X-ray and neutron 3D-ΔPDF to directly establish a quantitative defect model for YSZ. The signatures we observe on the shortest interatomic vectors, *i.e.* 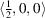 , 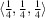 , 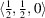 and 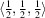 , enabled us to build a conclusive model for local correlations. The combination of the two different types of radiation is crucial for the quantitative analysis: the X-ray 3D-ΔPDF has a limited sensitivity to oxygen–oxygen correlations, while in the neutron 3D-ΔPDF the signatures of metal–metal correlations and oxygen–oxygen correlations overlap. The combined analysis allows the signatures to be disentangled and permits the quantitative analysis of local relaxation mechanisms. The shift directions we proposed from our analysis are in good agreement with previously reported relaxations from various experimental investigations and simulation efforts, the shift magnitudes we analyse are more pronounced than previously suggested. We consider our work as an important first step in the progress towards to a direct methods type analysis for diffuse scattering using 3D-ΔPDF methods.

Our study shows how a simple, direct interpretation of the 3D-ΔPDF leads to results that align well with established interpretations in literature, that involved complex modelling of the described correlations. In agreement with (Goff *et al.*, 1999 ▸) we find no evidence for interstitial metal atoms at 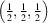 . We find strong evidence for double vacancy pairs along 〈½, ½, ½〉 (Bogicevic & Wolverton, 2003 ▸; Welberry *et al.*, 1993 ▸). Our analysis confirms oxygen displacements along 〈1, 0, 0〉 and metal displacements along 〈1, 1, 1〉 (Kaiser-Bischoff *et al.*, 2005 ▸), while we do not find direct evidence for oxygen displacements along 〈1, 1, 1〉 (Ishizawa *et al.*, 1999 ▸; Argyriou *et al.*, 1996 ▸) or metal displacements along 〈1, 1, 0〉 (Welberry *et al.*, 1993 ▸).

We present a simplistic static local-order model that describes the most local interatomic correlations well, but does not reproduce all the signatures observed in the data; in particular, the observed longer-range correlations are not described in the model presented here. One way of incorporating such longer-range interactions would be a big box modelling approach, *e.g.* a reverse Monte Carlo simulation: by fixing the directions and the magnitudes of the shifts to the values identified in our 3D-ΔPDF analysis, a reverse Monte Carlo simulation would be able to generate a model crystal that captures longer-range correlations that are not directly interpretable from the 3D-ΔPDF analysis. This in turn would provide a tool to identify longer-range interactions that are not present in the current model.

Our measurements are performed at ambient conditions; hence the model only captures correlations that are present at ambient conditions. However, for the technical application of YSZ as an oxygen ion conductor, the local order at elevated temperatures may be more relevant (Devanathan *et al.*, 2006 ▸; Kaiser-Bischoff *et al.*, 2005 ▸; Krishnamurthy *et al.*, 2004 ▸; Itoh *et al.*, 2015 ▸). For variable-temperature studies, the 3D-ΔPDF analysis as we present here facilitates a direct and quantitative comparison of correlations in real space as a function of temperature, providing a more complete picture than previously analysed selected sections of reciprocal space.

Another aspect that is of importance for technological applications is the interplay of compositional disorder and lattice dynamics. The picture presented here only describes a static local-order model and therefore does not cover the full picture of correlations at ambient conditions. The possible coupling of the local order to the lattice dynamics of a system is a particular challenge (Ziman, 1979 ▸; Snyder & Toberer, 2011 ▸). In YSZ the phonon anharmonicity at lower temperatures has been attributed to the defect structure and in turn to a higher vibrational entropy (Li *et al.*, 2015 ▸). A comprehensive, quantitative and reliable local-order model is needed for a complete understanding of the lattice dynamics in complex systems such as YSZ. In the future, the combination of temperature dependent energy discriminated diffraction experiments and supercell lattice dynamical calculations (Overy *et al.*, 2017 ▸; Schmidt *et al.*, 2022 ▸) based on our local-order model will enable a complete understanding of the structure and dynamics of such a complex locally ordered system.

## Supplementary Material

EDX measurements and additional plots. DOI: 10.1107/S205252062300121X/ra5126sup1.pdf

HTML files containing the 3D renderings of Figures 4 to 7 that can be turned and zoomed into. DOI: 10.1107/S205252062300121X/ra5126sup2.zip

Raw data of the ILL neutron diffraction experiment: https://dx.doi.org/10.5291/ILLDATA.5-13-277

## Figures and Tables

**Table 1:** Metal–oxygen bond lengths (Å) as a function of coordination number (CN) and sum of effective ionic radii

Bond	CN	MD simulation†	EXAFS	Sum of effective ionic radii¶
Y—O	6	2.267^*a*^	2.32‡	2.28
	7	2.267^*a*^	–	2.34
	8	2.340^*b*^	2.28§	2.40
Zr—O	6	2.117^*b*^		2.10
	7	2.117^*b*^	2.13‡/2.11§	2.16
	8	2.119^*a*^	2.11§	2.22

† Khan *et al.* (1998 ▸). The MD simulation distinguishes between oxygen vacancy NN to dopant ion and oxygen vacancy NNN to dopant ion. We interpret this in terms of coordination numbers. (*a*) NN dopant vacancy, (*b*) NNN dopant vacancy.

‡ Ishizawa *et al.* (1999 ▸).

§ Catlow *et al.* (1986 ▸).

¶ Shannon (1976 ▸).
