# Supplementary material for: Direct interpretation of the X-ray and neutron three-dimensional difference pair distribution functions (3D-ΔPDFs) of yttria-stabilized zirconia
Source: Acta Crystallogr B Struct Sci Cryst Eng Mater. 2023 Feb 24;79(Pt 2):138–47. doi: 10.1107/S205252062300121X (PMC10088481; doi:10.1107/S205252062300121X)
Supplement: Supplementary file 1 [file b-79-00138-sup1.pdf]

STRUCTURAL SCIENCE
CRYSTAL ENGINEERING
MATERIALS

Volume 79 (2023)

Supporting information for article:

Direct interpretation of the X-ray and neutron 3D-ΔPDFs of yttria-stabilized zirconia

Ella Mara Schmidt, Reinhard B. Neder, James D. Martin, Arianna Minelli, Marie-Hélène Lemée and Andrew L. Goodwin

1 Scanning Electron Microscopy (SEM) Energy Dispersive X-ray (EDX) analysis

A JEOL 6480 LV SEM equipped with an Oxford Instruments X-MAX80 SD X-ray detector and INCA X-ray analysis system was used to image the samples and perform the analysis using EDX. In this case the microscope was used in low vacuum mode, which a small amount of air is admitted to the specimen chamber to prevent the sample from charging. In this case a chamber pressure of 30 Pa was used and an accelerating voltage of 15 kV.

In total four spectra were taken from the specimen. The reconstructed spectrum is shown in Figure S1. Table S1 lists the results of the analysed spectra in atomic %. To estimate the composition we assume a composition $Zr_{1-x}Y_xO_{2-x/2}$ for our sample and consider the ratio of Y to Zr. With this we arrive at $x \approx 18\%$.

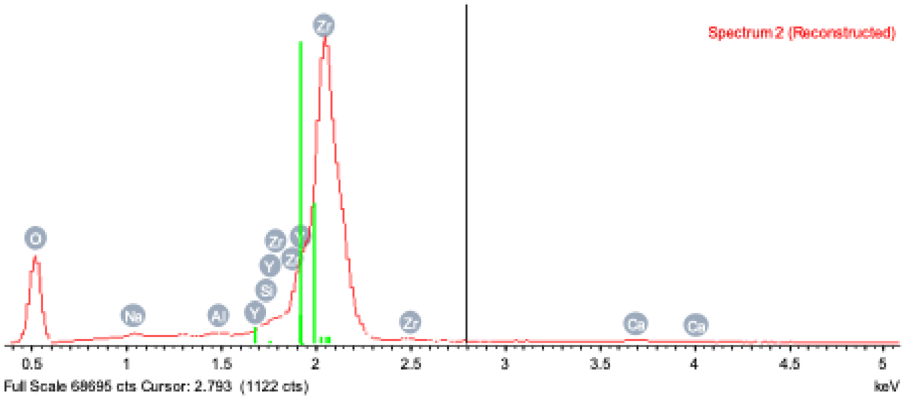

Figure S1: Reconstructed EDX spectrum.

	Y	Zr	Na	Al	Si	Ca	O
Spectrum 1	4.9	22.4	0.5	0.2	0.5	0.3	71.1
Spectrum 2	4.9	22.3	0.3	0.2	0.5	0.3	71.6
Spectrum 3	4.9	22.4	0.3	0.2	0.5	0.3	71.4
Spectrum 4	5.1	22.9	0.4	0.2	0.5	0.2	70.8
Mean	4.95(10)	22.50(27)	0.38(9)	0.20(0)	0.50(0)	0.28(5)	71.23(35)

Table S1: Results in atomic % from EDX measurement of the specimen.

2 X-ray measurement

The polished specimen is shown in Figure S2. Our measured X-ray 3D-ΔPDF shows a weak but clear signature at $\langle \frac{1}{2}, 0, 0 \rangle$ and therefore manifests that the oxygen displacements can be directly observed in the X-ray diffraction case and hence need to be included explicitly in any real-space and reciprocal-space modelling.

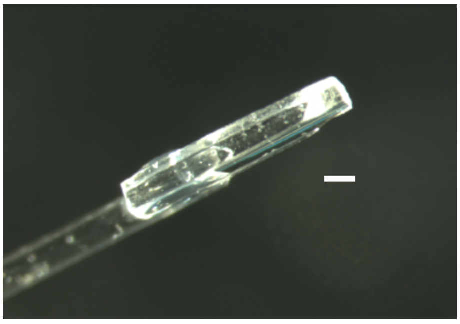

Figure S2: Polished crystal mounted onto glass fibre for X-ray diffraction experiments. Scale bar $\approx$ 200μm.

3 Maximum observable correlation length

The grid on which we reconstruct our experimental data is $501 \times 501 \times 501$ voxels in the volume $-10 \leq h, k, l \leq 10$. The voxel size in reciprocal space ($\Delta h = \Delta k = \Delta l = 0.04$) directly determines the maximum observable correlation length in real space. In our case we cannot observe correlations that are longer than 12.5 r.l.u.. For both the neutron and X-ray 3D-ΔPDF we do not observe correlations that are significantly longer than two unit cells, which is well below the limit given by the reciprocal space grid. In our reconstructed and also in the symmetry averaged diffraction patterns our Bragg peaks show a width of less then three voxels - for both the neutron and X-ray diffraction experiment. Assuming that three voxels is the upper limit of our experimental broadening, this reduces our maximum observable correlation length for the 3D-ΔPDF to $\approx$ 4 r.l.u., which is still significantly longer than the correlations we observe.

4 Comparison of the Data and the Model

Figures S3, S4 and S5 compare selected layers fo the calculated diffuse scattering and the 3D-ΔPDF from the simplistic atomistic model to the experimentally obtained data.

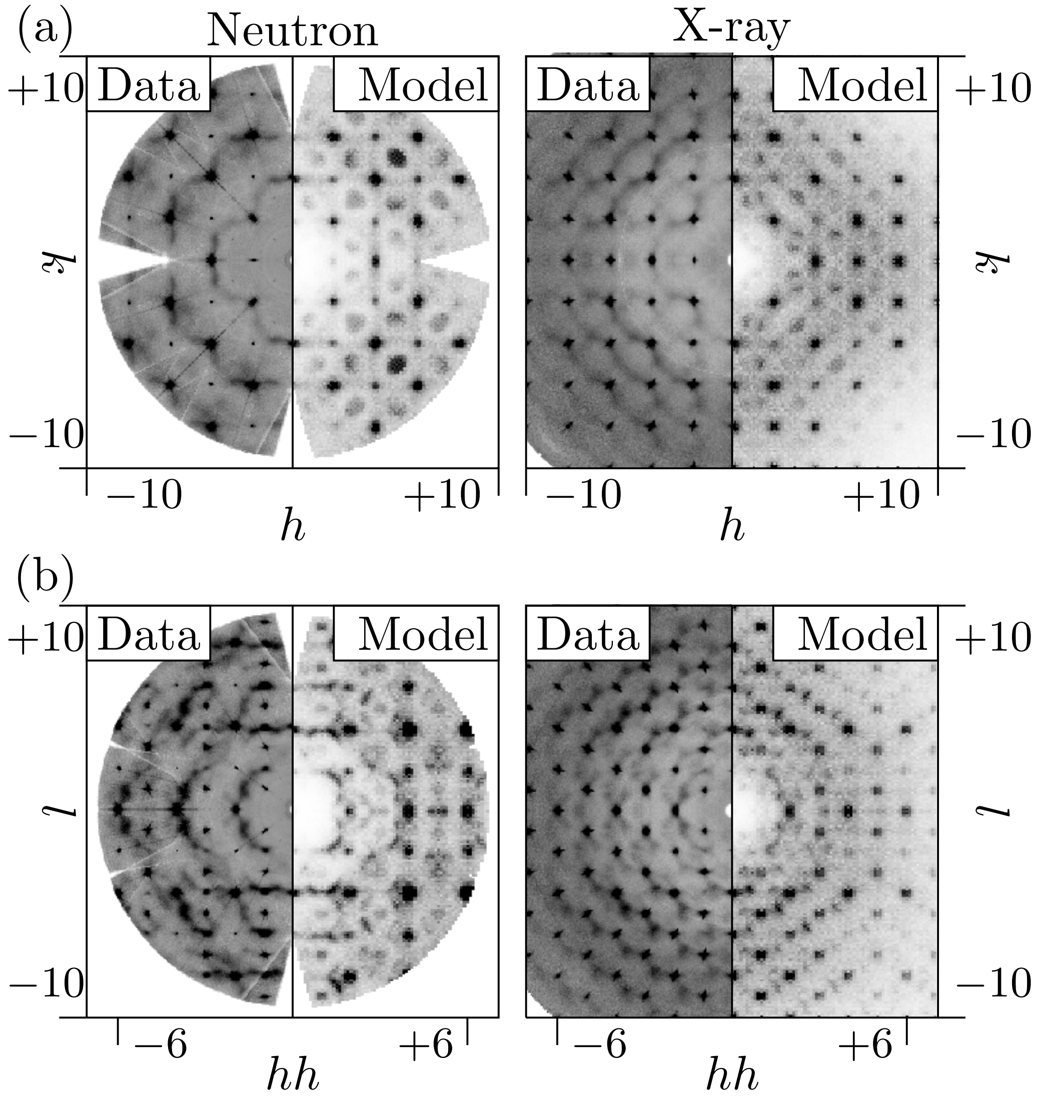

Figure S3: Reconstructed and symmetry averaged diffuse scattering of YSZ obtained from neutron (left) and X-ray (right) diffraction experiments compared to the results of modelling. (a) $hk0$-layer, (b) hhl-layer.

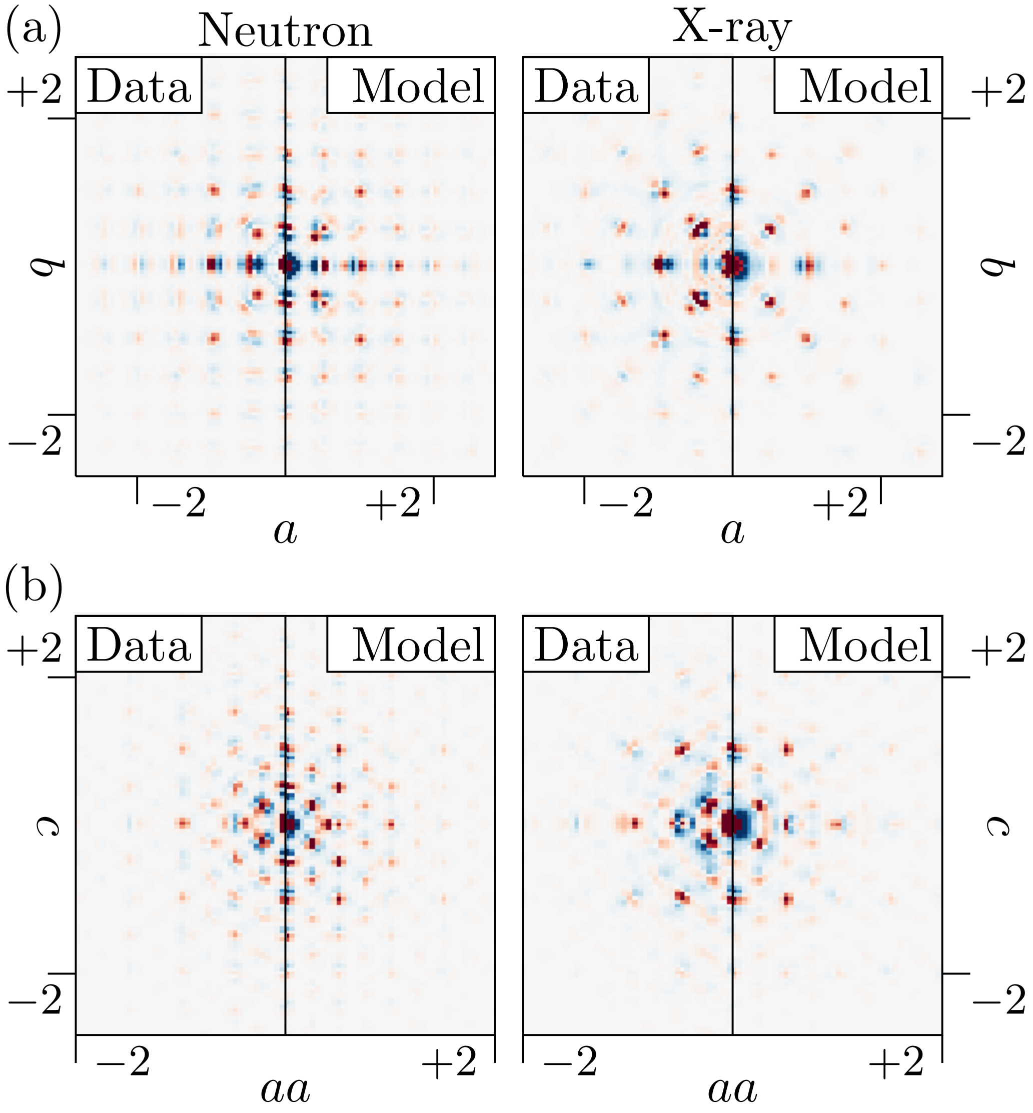

Figure S4: 3D-ΔPDF maps of YSZ obtained from neutron (left) and X-ray (right) diffraction experiments compared to the corresponding maps from our model calculations. (a) $ab0$-layer, (b) aac-layer. Positive intensities in red, negative intensities in blue.

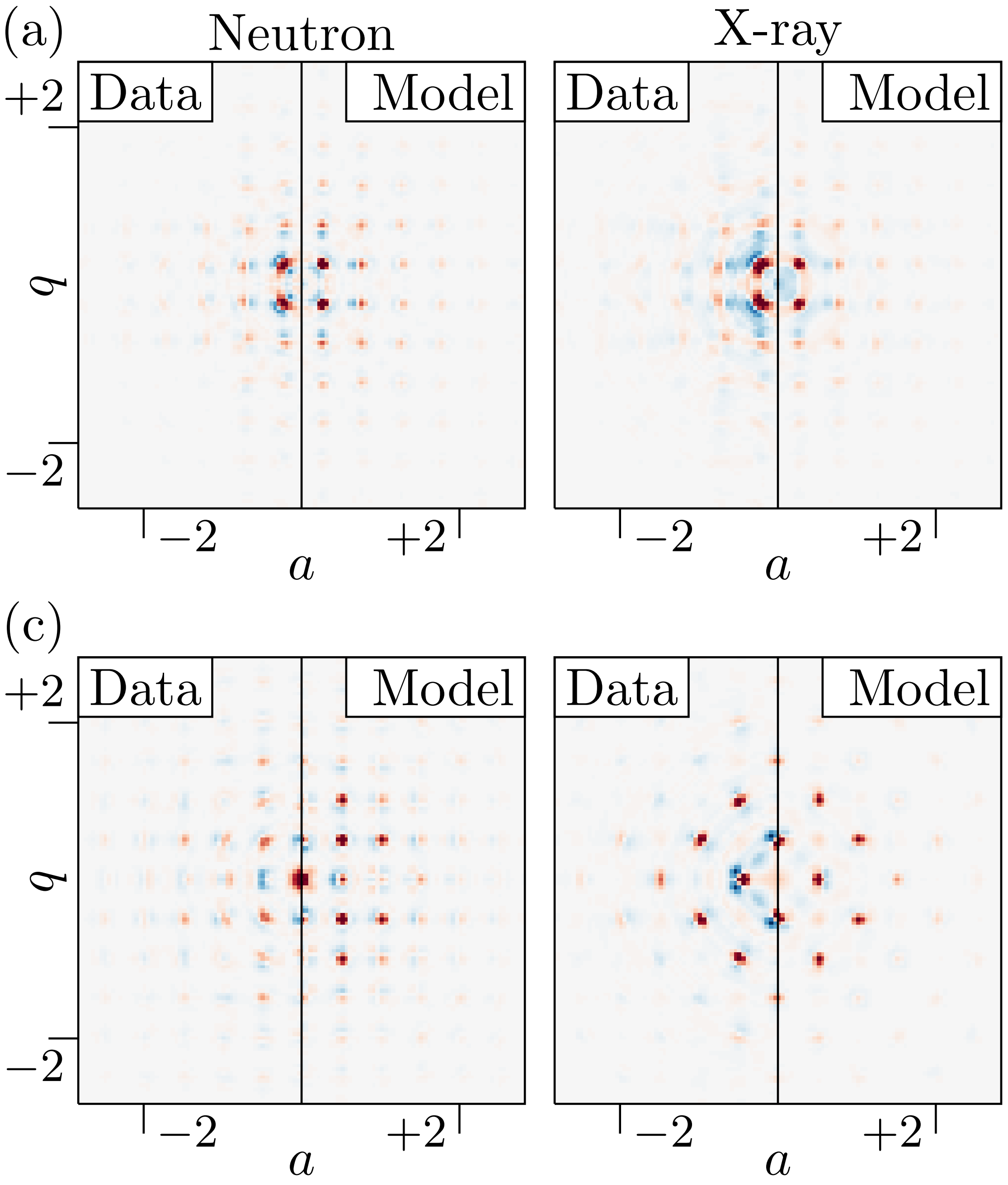

Figure S5: 3D-ΔPDF maps of YSZ obtained from neutron (left) and X-ray (right) diffraction experiments compared to the corresponding maps from our model calculations. (a) $ab0.25$-layer, (b) $ab0.5$-layer. Positive intensities in red, negative intensities in blue.
